# Primary metabolic processes as drivers of leaf ageing

**DOI:** 10.1007/s00018-021-03896-6

**Published:** 2021-07-19

**Authors:** Aakansha Kanojia, Deny K. Shrestha, Paul P. Dijkwel

**Affiliations:** 1 Center of Plant Systems Biology and Biotechnology, Ruski 139 Blvd., Plovdiv, 4000 Bulgaria; 2 School of Fundamental Sciences, Massey University, Private Bag 11222, Palmerston North, New Zealand

**Keywords:** System biology, Ageing, Primary metabolism, DNA repair, Reactive oxygen species, Molecular genetics

## Abstract

Ageing in plants is a highly coordinated and complex process that starts with the birth of the plant or plant organ and ends with its death. A vivid manifestation of the final stage of leaf ageing is exemplified by the autumn colours of deciduous trees. Over the past decades, technological advances have allowed plant ageing to be studied on a systems biology level, by means of multi-omics approaches. Here, we review some of these studies and argue that these provide strong support for basic metabolic processes as drivers for ageing. In particular, core cellular processes that control the metabolism of chlorophyll, amino acids, sugars, DNA and reactive oxygen species correlate with leaf ageing. However, while multi-omics studies excel at identifying correlative processes and pathways, molecular genetic approaches can provide proof that such processes and pathways control ageing, by means of knock-out and ectopic expression of predicted regulatory genes. Therefore, we also review historic and current molecular evidence to directly test the hypotheses unveiled by the systems biology approaches. We found that the molecular genetic approaches, by and large, confirm the multi-omics-derived hypotheses with notable exceptions, where there is scant evidence that chlorophyll and DNA metabolism are important drivers of leaf ageing. We present a model that summarises the core cellular processes that drive leaf ageing and propose that developmental processes are tightly linked to primary metabolism to inevitably lead to ageing and death.

## Introduction

The plant kingdom includes some of the longest living species on earth, with bristlecone pine at a demonstrated lifespan of several thousand years and a clonally reproducing seagrass estimated at a minimum of 80,000 years [1, 2]. However, much of the living tissue of each plant ages over a much shorter time, often in response to seasonally driven cold or dry periods. In deciduous trees, this is most spectacularly seen in autumn, when leaves change colour and the subsequently rapid fall of leaf foliage. In fact, it has been proposed that the short life cycle of leaves and other plant tissue is at the basis of the extreme longevity of the whole organism [3, 4]. Nevertheless, since it is difficult to study whole plant ageing in trees, which live many times longer than the researchers studying the tree, plant leaves are often chosen to study ageing. Here, we review literature on leaf ageing and ageing of short-lived annual plants. The conclusions drawn from such studies, therefore, do not necessarily apply to long-lived plants.

We consider that leaf ageing encompasses the whole lifespan of a leaf, i.e., starting from initiation and ending with the process called leaf senescence. Leaf senescence is the most studied aspect of leaf ageing and involves a highly regulated and complex process requiring a vast set of genes that interact through multiple signalling pathways [5, 6]. Senescence of individual leaves can take place throughout plant development, but at the end of monocarpic plants’ lifes, all leaves senesce at the same time [7]. Senescence of a leaf begins when the photosynthetic rate drops below a certain threshold; therefore, the leaf no longer contributes to carbon fixation [8]. De-greening of the leaves, due to chlorophyll degradation, is a visible indicator of leaf senescence [9]. Since, chlorophyll, protein, and membrane degradation occur during senescence, the levels of these parameters are used as markers in the study of senescence progression [10, 11]. The phenomenon of leaf senescence also includes other cellular catabolic processes such as lipid peroxidation and degradation of proteins, carbohydrates, RNA, and DNA [8, 12]. The dismantling of cellular components during the senescence process releases a considerable amount of nutrients, which are then remobilised to the growing parts of the plant [13, 14]. Thus, leaf senescence is an active and destructive process but essential for development of actively growing organs such as new leaves, seeds, and buds that need to survive through winter [8, 15–17].

Recent technological and computational ‘omics’ advances have allowed researchers to take a more holistic approach to studying the regulation of senescence processes. However, the events leading to the senescence process are poorly understood. Here, we review computational studies and analyse outcomes relevant to leaf ageing, to refine hypotheses that describe processes that may drive leaf ageing. While computational studies excel at sifting through large datasets to identify potential regulatory hubs and processes, hypotheses developed through these studies often need to be tested using genetic methods that perturb those processes, like gene knock-out or overexpression studies. Thus, we subsequently examine evidence that supports or rejects computer-based hypotheses. Finally, we summarise and highlight general mechanisms of leaf ageing, thereby largely overlooking ageing processes that are species-specific or that affect the onset of senescence due to reproduction or a changing season in monocarpic and deciduous plants, respectively. Nevertheless, since ageing is such a universal process, we expect that general mechanisms are conserved throughout the plant kingdom.

## Systems biology approaches of ageing suggest primary metabolic processes as major age-regulating factors

Various ‘omics’ studies in the field of plant ageing have helped develop hypotheses to explain the complex plant ageing system [19, 20]. Nevertheless, these studies mainly illustrate changes in biological pathways during natural or stress-induced senescence [18, 20]. Genomics research has contributed to understanding the genetic composition, function, and potential mechanism of the genes that regulate ageing and senescence in plants [20]. Transcriptomics has provided extensive information about the gene expression patterns in ageing tissue and while it cannot accurately predict the metabolome, it provides background information for metabolomics studies [21–23]. Metabolomics, while generally less comprehensive, may potentially be more informative than genome and gene expression studies because metabolites constitute the end-product of all cellular functions, and thus take into account complex enzymatic pathways where enzyme activity is modulated by regulatory proteins and the metabolome itself.

First, we review multi-omics studies of ageing leaves, which suggest that changes in core cellular processes, like chlorophyll, amino acid, sugar, DNA, and reactive oxygen species (ROS) metabolism, are an outcome—or may be drivers—of plant ageing.

### Reactive oxygen species

Well over half a century ago, Harman (1956) hypothesised that oxygen radicals, as by-products of endogenous metabolism, inflict oxidative stress in all living things, leading to loss of functional efficiency in multiple cellular processes and ultimately to ageing [24]. On top of metabolic processes present in all eukaryotes, photosynthetic activity in plants adds considerably to ROS production [25, 26]. At moderate levels, ROS help to modulate various growth and developmental processes [27], but an excess of ROS is toxic and causes damage to various cellular organelles and components [28, 29]. It is well reported that in plants, ageing is accompanied and accelerated by the overabundance of ROS [27, 30–33].

While relatively few transcriptomics reports have specifically addressed the ROS-ageing relationships, some of these studies indicate that cellular oxidative stress increases with plant and leaf age. For instance, leaves of 3000-year-old *Platycladus orientalis* had higher ROS levels together with the upregulation of genes involved in ROS production and stress responses than the leaves of a 20-year-old plant of the same species [34]. Likewise, age-dependent transcriptomes of *Arabidopsis* leaves showed that the expression of genes associated with oxidative stress were elevated in leaves before the completion of leaf growth and senescence initiation [6, 35]. An analysis of the *Arabidopsis* leaf transcriptome during ageing also showed enrichment of abiotic stress-responsive genes in matured leaves before the onset of senescence [19]. The upregulated oxidative stress genes include several senescence associated genes (*SAGs*) and genes that encode mitogen activated protein kinases (MAPKs), WRKY and NAC transcription factors, that function to limit oxidative stress. However, these studies did not differentiate between upregulation of genes solely to limit oxidative stress, or upregulation to control and allow an age-induced increase in oxidative stress.

### Chlorophyll metabolism and photosynthesis

Chlorophyll is an essential component of photosynthesis in plants and, therefore, it can be expected that biosynthesis of photosynthesis components is tightly coupled to leaf development and that such biosynthesis is ceased once senescence starts. However, since sugar status and the ability to export sugars out of the leaf can regulate leaf senescence, photosynthetic capacity may play a more direct role in the regulation of ageing and induction of leaf senescence [36–38]. Consistent with that idea, Breeze et al. found a significant switch in gene expression of photosynthesis-related genes, prior to the onset of senescence: reduced expression of two Rubisco small subunit genes that encode critical enzymes for CO_2_ fixation in the Calvin cycle was observed five days earlier than visual signs of leaf senescence [6]. Furthermore, the expression of genes involved in chlorophyll degradation, *SGR1*, *SGR2*, *NYC1* (Chlorophyll b reductase) and *PaO* (Pheophorbide a oxygenase) increased before leaf tip yellowing was observed. Moreover, transcriptomic studies performed in Switchgrass cultivars showed that changes in expression of chlorophyll biosynthesis and degradation-related genes occurred weeks before the visual onset of leaf senescence [39].

### Primary metabolites

Biosynthesis of sugars and amino acids is tightly coordinated with photosynthesis to meet the plant’s demand for energy required during development [40, 41] and must therefore be highly regulated. Sugars are the primary products of photosynthesis and are transported from source tissues to the sink organs via several sugar transporters [42]. In light, sugars are converted to starch, which is remobilised at night to support metabolism and growth of plants [43]. In a recent study, analysis of primary metabolites in progressively ageing *Arabidopsis* leaves showed that 14 sugar metabolites, including fructose, glucose, maltose, mannitol, galactose, trehalose, and other unidentified sugars that were high in young expanding leaves, decreased gradually during leaf expansion, and well before the onset of leaf senescence [35]. A similar pattern of reduction in sugar metabolites before the initiation of senescence was observed in comprehensive metabolomics studies performed on *Nicotiana tabacum* (tobacco) leaves during five developmental stages [44].

Several studies have found that sugar starvation due to limited photosynthesis activity initiates the onset of leaf senescence [37, 45–48] and gene expression involved in carbohydrate metabolism decreased before the onset of senescence [6]. Moreover, carbohydrate biosynthesis-related genes were upregulated in leaves during the growth to maturation stage but downregulated in the maturation to senescence stage [19].

**Fig. 1:**
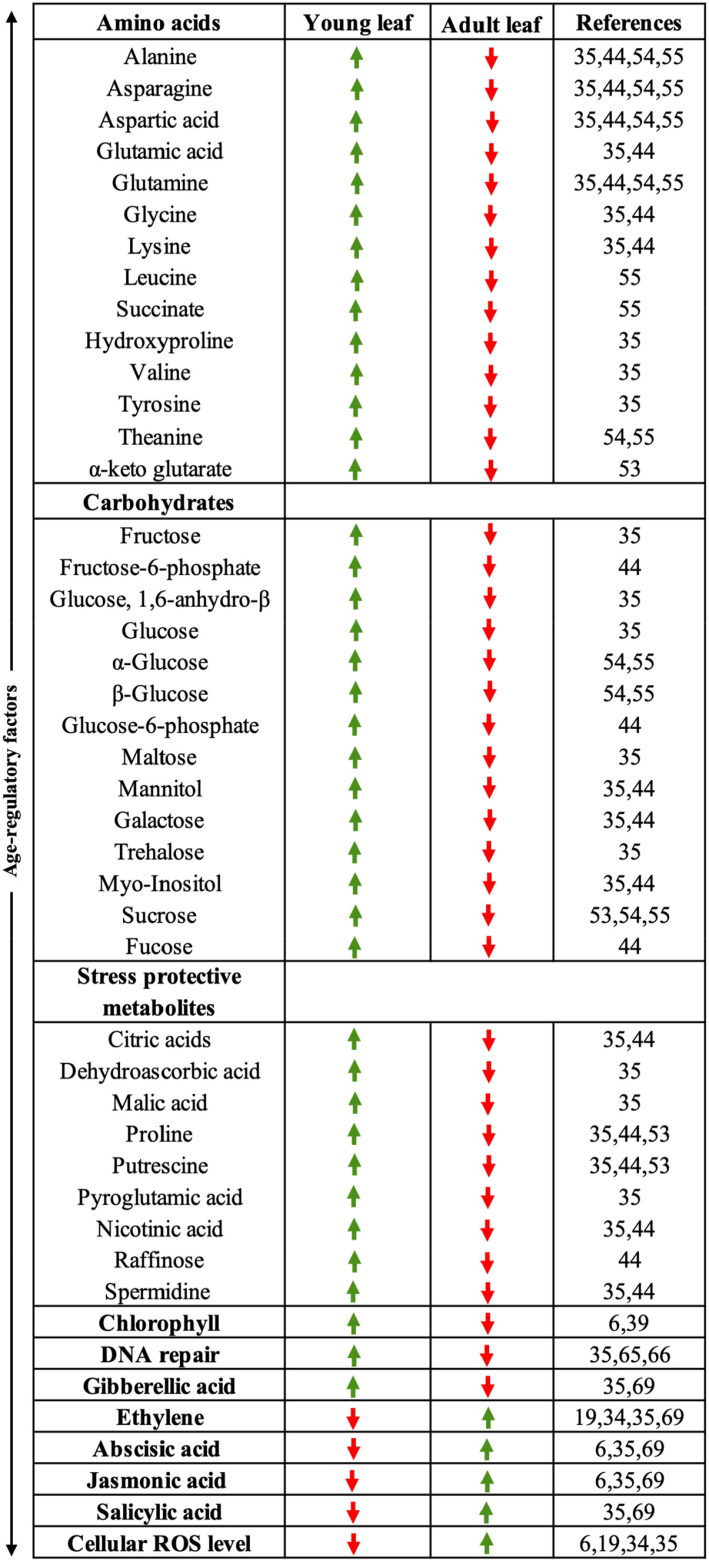
Changes in metabolism in different aged leaves. Primary metabolic changes in young and adult leaf shown by transcriptomic and metabolomic studies. Green and red arrows indicate relative abundances of metabolites to be higher or lower, respectively, as compared between young and old leaves

Many primary metabolites have protective properties to help plants tolerate oxidative stress [49–52]. Crucially, the stress metabolites proline, putrescine, pyroglutamic acid, spermidine, citric acid, raffinose, and dehydroascorbic acid are well reported to play central roles in stress tolerance and were found to decrease during leaf maturation, before the onset of senescence (Fig. 1) [35, 44, 53]. Other basic metabolic changes that regulate senescence were reported in young and mature but not yet senescing leaves of both perennial and annual plants: for instance, downregulation of genes that are involved in amino acid biosynthesis and a decrease in the level of amino acids in developing *Medicago sativa* (alfalfa) and *Arabidopsis* leaves [6, 35, 54]. Likewise, in a recent study, age-related differences were observed in metabolites of leaves collected from perennial tea crop *Camellia sinensis* plants aged 8 and 25 years old [55]. Compared to 8-year-old samples, tea leaves collected from older plants showed lower alanine, glutamine, theanine, asparagine, leucine, succinate amino acid and sucrose, α-glucose and ß-glucose sugar abundances [55]. In this case, the leaves were of similar age and this suggests that primary metabolites may change not only with leaf age, but also with plant age in perennial plants. Thus, the age-related decrease of many primary metabolites may be a manifestation of leaf ageing processes, or may contribute to ageing.

### DNA repair

Living organisms, including plants, are protected against DNA damage caused by endogenous and exogenous factors by means of DNA repair mechanisms [56, 57]. In plants, unrepaired DNA damage can lead to early onset of senescence [58]. Also, in humans, the accumulation of DNA damage over time is linked to early ageing [59, 60]. Multiple studies suggest that accumulation of unrepaired DNA can gradually reduce cellular functions and accelerate ageing in a variety of species, including *Saccharomyces cerevisiae*, *Caenorhabditis elegans,* and *Drosophila melanogaster* [61–64]. Thus, there is evidence across kingdoms that links DNA damage with early ageing. In *Arabidopsis*, DNA repair efficiency was higher in young leaves than in adult leaves [65] and *Arabidopsis* transcriptomic data of expanding leaves also showed a strong negative relation between expression of genes involved in DNA repair mechanisms and age [35].

**Fig. 2:**
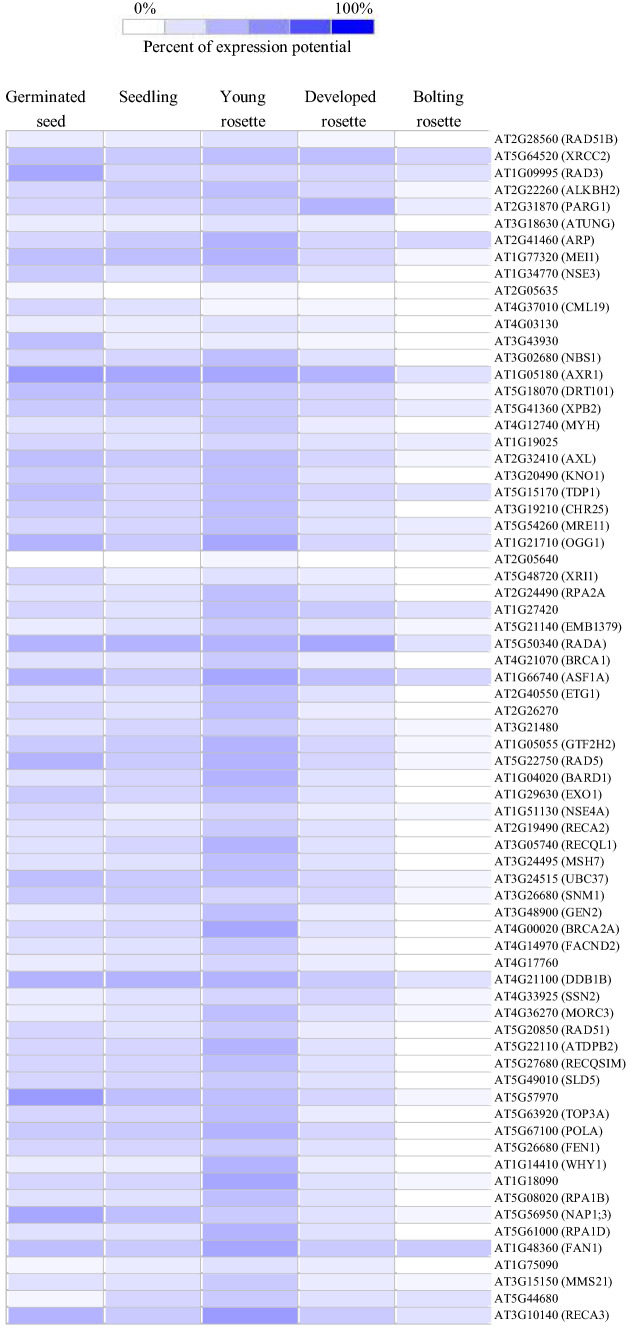
Expression patterns of DNA repair-related genes, obtained from TAIR, in *Arabidopsis* tissue of increasing age, plotted by the Genevestigator development tool. Wild type *Arabidopsis* mRNA dataset was chosen to generate the heat map. In Genevestigator, the colours (values) are normalised to the expression potential of each gene and the expression potential indicated for a given stage of development is the average of expression of all samples annotated. The colour scale bar from white to blue shows the percentage of the gene expression potential. The darkest blue represents the maximum level of expression and white colour represents the minimum level of expression

To further asses the possible role of DNA repair in ageing, we obtained a list of DNA repair-related genes from TAIR (https://www.Arabidopsis.org/) and visualised the gene expression patterns in *Arabidopsis* tissues from germination to the rosette bolting stage using the Genevestigator development tool (https://genevestigator.com/gv/; [66]). The heat map in Fig. 2 shows a very strong correlation between rosette development and expression of DNA repair genes, with the highest expression in young rosette tissues, and the lowest during the bolting stage. These results too indicate that DNA repair mechanisms are developmentally regulated, and therefore, maintenance of genomic integrity may decrease as the leaves age. A confounding factor is that during leaf development, the cell cycle completes, resulting in a reduced requirement for DNA repair mechanisms [65]. Nevertheless, DNA damage will still occur and the combination of increased oxidative stress during leaf expansion [35] and reduced DNA repair may result in decreased genome stability and ageing. Moreover, these results are solely based on *Arabidopsis* tissues.

### Hormones

Plant hormones have a wide range of functions during all phases of development and thus also during ageing. The hormones ethylene, abscisic acid (ABA), jasmonic acid (JA), and salicylic acid (SA) have been described to initiate plant ageing, while cytokinin, auxin, and gibberellic acid (GA) are believed to delay plant ageing [17, 67, 68]. *Arabidopsis* leaf transcriptome analysis is in general agreement with this [6, 35, 69], although a more or less equal percentage of cytokinin and auxin-related genes were up and downregulated in expanding leaves [35, 69]. Fifteen out of 18 GA biosynthesis-related genes decreased in *Arabidopsis* leaves from the expanding to the senescence phase, which suggests that active forms of GA decline as the leaf ages [69]. Ethylene is known as a master regulatory hormone in inducing leaf senescence [70–72]. Chang et al. showed that the expression of ethylene biosynthesis-related genes was increased in an old *Platycladus orientalis* tree as compared to a young one [34], and in *Arabidopsis*, all the ethylene-responsive genes were upregulated in mature leaves as compared to young leaves [35]. Likewise, during late developmental stages, i.e., from maturation to senescence, *Arabidopsis* leaf transcriptomes showed the enrichment of genes associated with ethylene biosynthesis and signalling [19, 69]. Moreover, the majority of genes involved in the biosynthesis and signalling of ethylene, SA, ABA, and JA, increased during *Arabidopsis* leaf expansion [35]. Also, the expression of a vast majority of JA, ABA, and SA biosynthesis-related genes increased in *Arabidopsis* from the rapid expansion leaf stage to the leaf senescence stage, suggesting a role for these hormones in leaf ageing [69]. Thus, leaf transcriptome findings support the well-known roles of ethylene, SA, ABA, and JA in ageing.

### Changes in metabolic signatures during leaf ageing

**Fig. 3:**
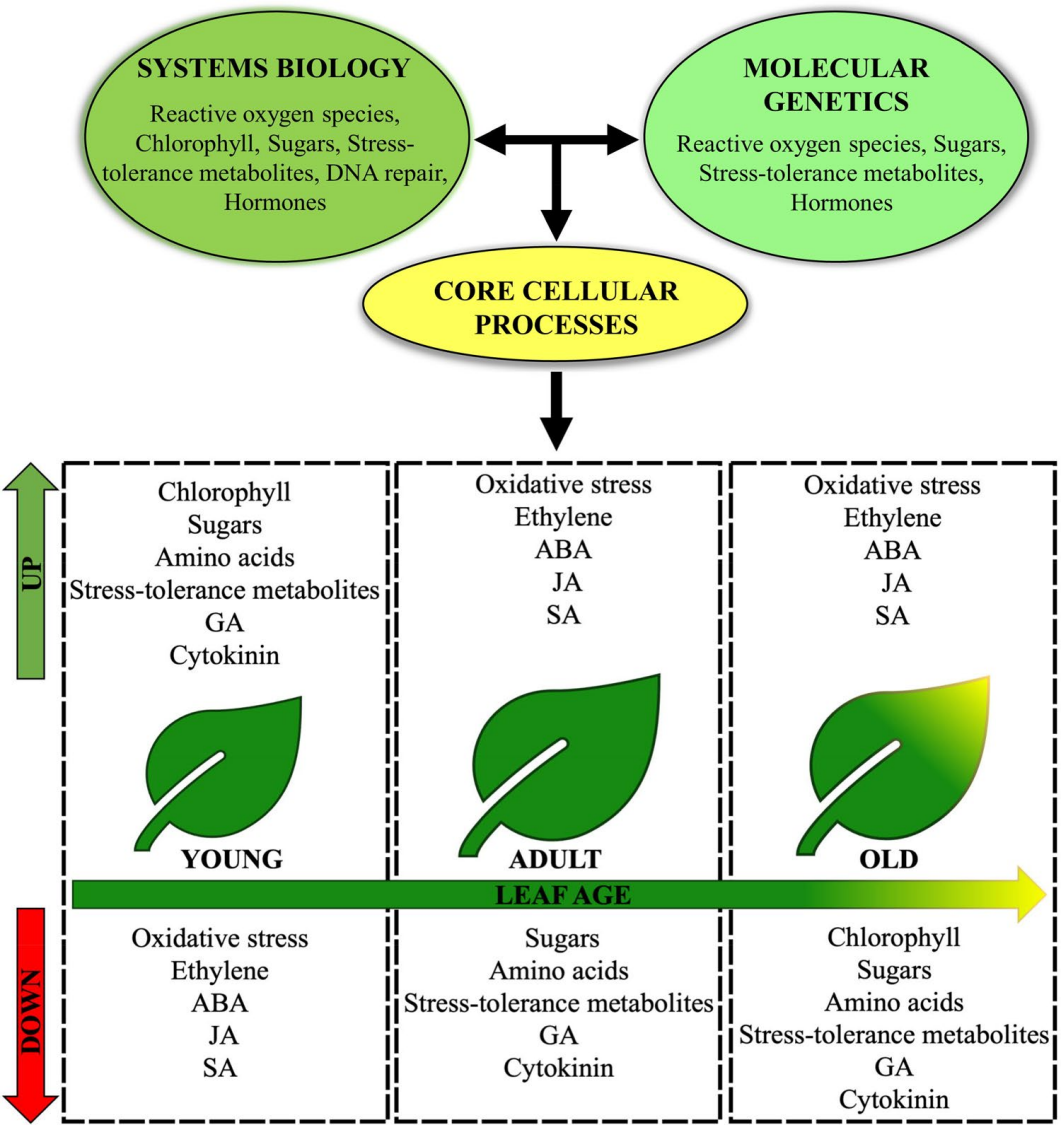
Core cellular processes contribute to plant ageing. A combined systems biology and molecular genetic approach suggests that changes in core cellular processes that occur during leaf ageing are regulating ageing. The model shows that the elevated growth-promoting metabolites in young leaves decrease in mature and old leaves, whereas the lower levels of cellular oxidative stress and stress-responsive hormones elevate as the leaf ages. *GA* gibberellic acid, *ABA* abscisic acid, *JA* jasmonic acid, *SA* salicylic acid

Transcriptomic and metabolomic studies of developing leaves show a strong correlation between core cellular processes and ageing (Figs. 1 and 3). During early leaf development, core cellular processes like chlorophyll biosynthesis and DNA repair accompany growth, while the abundance of sugars, amino acids, GA, and metabolites that provide stress tolerance, deliver the means to sustain active growth. Then, as the leaf matures, an increase in cellular oxidative stress and senescence-inducing hormones coincides with a marked reduction in chlorophyll biosynthesis, DNA repair and abundance of sugars, amino acids, and stress-tolerant metabolic activities. Thus, Fig. 3 illustrates the patterns of core cellular processes in young and adult leaves, underpinning their potential role as major age-regulatory factors.

However, the transcriptomic and metabolomic work unveiled correlative, rather than causal relationships. For example, these studies do not distinguish between the hypotheses that chlorophyll biosynthesis, and the drop thereof, drives ageing or, that ageing processes drive chlorophyll anabolic and catabolic processes. Fortunately, ample molecular genetic studies, dating back decades and more current ones, have shown causality. Below we aim to test hypotheses suggested by above-described work using mutant and physiological studies that perturb primary metabolism pathways.

## Molecular genetic approaches confirm the role of core cellular processes in leaf ageing

Gene knock-out and overexpression studies are extremely powerful at demonstrating causal relationships and may provide the best proof for pinpointing genes and processes involved in the regulation of ageing. Nevertheless, knock-out of essential genes involved in development or primary metabolism can render a plant unviable, and leaky mutations may not exhibit a clear phenotype. Moreover, genes that possess multiple functions, which is often the case for those involved in hormone biosynthesis and response, may affect plants during early growth, and this in turn may cause secondary developmental changes that wrongly connect the gene knock-out to the regulation of ageing. Overexpression of genes can cause ectopic phenotypes, which may mask or alter an ageing phenotype. Conditional knock-down or overexpression studies may solve these issues, but it is difficult to create complete knock-outs at the right time and tissue. Therefore, to conclude that a metabolic pathway regulates ageing, multiple studies—preferably using various gene perturbations in the process—should be performed. Below we describe combined molecular genetic evidence in core cellular processes to conclude whether they regulate, or correlate with ageing.

### ROS metabolism drives ageing

Since ROS play important roles in signalling and their abundance is well controlled using multiple scavenging pathways [29, 73, 74], a direct correlation between ROS and ageing has been difficult to establish. Nevertheless, a good number of studies in the model plants *Arabidopsis* and *Oryza sativa* (rice) have helped to understand the involvement of ROS in plant ageing. Woo et al. showed that oxidative stress tolerance is associated with leaf longevity in the *Arabidopsis* *oresara* mutants: *ore1 *(*NAC Transcription factor*), *ore3* (*EIN2*) and *ore9* (*F-Box protein*) [75]. However, the reason for the oxidative stress tolerance in those mutants is elusive as those mutants did not show increased activity of antioxidants compared to wild type. The *onset of leaf death5* (*old5)* early ageing *Arabidopsis* mutant showed a higher respiration rate, along with higher oxidative stress, well before the onset of senescence [76]. Single and double mutants of *cat2*, *cat3,* and *cat2*/*cat3* did not show any significant differences during development, with not much reduction in ROS accumulation [77]. This suggests that there are other ROS scavenging channels that compensate the ROS detoxification in the absence of catalases in those mutant plants. A well-studied ROS regulatory gene encodes the H_2_O_2_-induced NAC Transcription factor (JUB1), which functions to reduce H_2_O_2_ production [78–81]. Overexpressed lines of *JUB1* in *Arabidopsis* had a diminished intracellular H_2_O_2_ production, thereby delaying bolting and plant ageing. In contrast, the *jub1-1* knock-out mutant showed early bolting and early ageing with lower tolerance to abiotic stress [79]. In rice, inactivation of *UDP-N-acetylglucosamine pyrophosphorylase 1* (*OsUAP1*), *Nicotinate Phosphoribosyl transferase* (*OsNaPRT1*), *O-methyltransferase* (*OsMTS1*), *reactive oxygen species-sensitive leaf senescence1* (*RLS1*), and *premature senescence of flag leaf* (*psf,* encoding superoxide dismutase), all showed early leaf ageing as evidenced by premature induction of a leaf senescence phenotype, that correlated with an increase in cellular ROS, hypersensitivity to oxidative stress, or a lower activity of ROS-scavenging enzymes [82–86].

Thus, consistent with the transcriptomic studies, considerable genetic evidence corroborates the relationship between ROS and plant ageing.

### Chlorophyll metabolism can affect senescence

Photosynthesis captures energy and transforms it into sugars and in the process produces ROS [73, 87]. Thus, photosynthesis could contribute to regulating ageing in different ways. A reduction in chlorophyll biosynthesis may decrease the ability of an ageing cell to maintain its chloroplasts and therefore decrease photo assimilate production. In addition, photosynthesis-related ROS production may contribute to ageing and the degradation of chlorophyll involves the production of highly phototoxic intermediate degradation products [88].

*Arabidopsis*, rice and *Medicago truncatula* mutants that stay green for longer than the wild type have been isolated, thus suggesting that ageing and senescence may be delayed. Some of these mutations were found to affect chlorophyll degradation, for example, *pph -1* (*pheophytinase*), *nyc1* (*chlorophyll b reductase* or *non-yellow coloring1*), *sgr* (*staygreen*) and *nol* (*NYC1-LIKE*) [89–97]. However, in these mutants, photosynthetic activity was not altered, and cellular damage not delayed as compared to the wild type. Thus, such cosmetic (non-functional) mutants are useful for the study of chlorophyll degradation but not for studying leaf ageing, as the retention of greenness in these mutants does not lead to increased leaf longevity.

However, transcription factors ANAC072, ANAC046 and ANAC016 were found to regulate chlorophyll degradation and senescence by binding to the promoter sites of *NONYELLOWING1* (*NYE1*, also known as *SGR1*) and activating the transcription of these genes [98–100]. The loss of function mutant *anac072-1* showed a staygreen phenotype with slower retardation of photosynthetic efficiency than wild type, whereas the overexpression mutant *anac072-2* exhibited an opposite phenotype [98]. Furthermore, various staygreen mutants of durum wheat maintained photosynthetic competence for longer than the control plant [101]. Thus, these studies show that some genes that regulate chlorophyll degradation, regulate leaf senescence as well. However, there is little evidence for a role of chlorophyll metabolism in ageing processes other than senescence.

### Primary metabolism affects ageing

Sugars play critical roles during all aspects of plant growth [102, 103] and mutations that cause altered sugar metabolism are likely to affect aspects of plant growth, including ageing, making it difficult to pinpoint a direct effect of sugars on ageing. Nevertheless, in *Arabidopsis*, sugar signalling has been shown to affect an important ageing switch in *Arabidopsis*, the transition from juvenile to adult phase [104]. A lower sugar content led to increased abundance of *microRNA156* (*miR156*), thereby restricting the transition of juvenile leaves to adult leaves, while an exogenous supply of sucrose promoted adult leaf growth by repressing the transcription of *miR156* genes [104, 105]. Sugars were also convincingly demonstrated to affect another aspect of ageing: the timing of leaf senescence. Lower sugar abundances generally delay senescence. In artificial media, high glucose in combination with low nitrogen can induce senescence [37, 47]. Wang et al. showed that in the rice *premature leaf senescence2* (*pls2*) mutant accumulation of sucrose and starch grains, in chloroplast containing cells, reduced expression of photosynthesis-related genes in combination with premature leaf senescence [106]. Disruptions of genes that sense or regulate sugars also affect the timing of senescence. Mutations in *Arabidopsis* *Hexokinase-1* (*HXK-1*) decreased hexose abundance and delayed leaf senescence [47, 107], while its overexpression in *Solanum lycopersicum* (tomato) induced leaf senescence [108]. Furthermore, an orthologue of *sucrose nonfermenting1* (*SNF1*)/*Snf1-related kinase1* (*SnRK1*) acts as a sensor for starvation or low glucose condition [109]. Transgenic *Arabidopsis* with overexpressed *KIN10* (*SnRK1* homologue) showed longer leaf longevity and increased tolerance to nutrient deficiency. Likewise, early leaf senescence was reported in plants with reduced levels of *KIN10* and *KIN11* [110]. Other studies, some of which used dark-detached leaves, showed that sugar starvation may induce leaf senescence and external application of sugars can retard the senescence process [48, 111–113]. Thus, while sugars regulate ageing, the molecular basis of this mechanism is poorly understood.

Other metabolites have also been linked to plant ageing, especially those that are involved in ROS and stress regulation. The polyamines putrescine, spermidine, and spermine and γ-amino butyric acid (GABA), are all associated with the regulation of ageing in plants. In *Arabidopsis*, the GABA transaminase knock-out mutant (*pop2*) showed higher ROS accumulation, early flowering and early onset of leaf senescence [114]. Constitutive overexpression of *arginine decarboxylase* (*ADC*) and *polyamine uptake transporters* (*PUT*) caused accumulation of putrescine and spermidine, respectively, in leaves and displayed a delayed flowering phenotype [115, 116]. Furthermore, the *Polyamine Oxidase 4* (*pao4*) mutant had increased spermine and reduced spermidine contents, and showed a delayed onset of dark-induced leaf senescence. The *pao4* mutant was also associated with reduced H_2_O_2_ and lipid peroxidation levels and increased abundance of redox regulating metabolites [117]. In rice, the *spotted leaf32* (*spl32*) mutation disrupted the gene encoding ferredoxin-dependent glutamate synthase (Fd-GOGAT), which is involved in biosynthesis of glutamate. This mutant showed lower glutamate accumulation combined with higher levels of H_2_O_2_ and other major amino acids and these changes coincided with early leaf senescence [118].

These studies suggest that changes in contents of many primary metabolites regulate ageing, some of those likely by means of altering ROS levels.

### Do DNA repair processes regulate ageing?

Transcriptome analyses provide a strong argument that DNA repair mechanisms are regulating ageing in plants. Also, based on extensive research in other eukaryotes, decreased DNA repair can be expected to limit the lifespan of plants. However, little firm evidence confirms this hypothesis. Two decades ago, Riha et al. studied *Arabidopsis* telomerase-deficient T-DNA lines of *AtTERT* and while early knock-out generations appeared phenotypically normal, later generations displayed massive chromosomal instability [119]. The loss of telomeres impaired growth, but the leaves of the mutant remained green and metabolically active for months. It was also shown that *Arabidopsis ddm1* (*decrease in DNA methylation 1*) mutant plants exhibited delayed ageing with reduced DNA repair capabilities and susceptibility to salt stress [120, 121]. These studies support a link between DNA repair and ageing in plants. On the contrary, mutations in the *rad51b*, *rad51d,* and *xrcc2* genes, which are important for DNA repair displayed normal vegetative and reproductive growth [122], and we did not detect an ageing phenotype in the *recQ4A* mutant that displayed genomic instability, despite actively looking for it [123].

Recently, a comprehensive study performed by Li et al., separately knocked out 13 *Arabidopsis* genes involved in DNA repair and specifically looked for leaf ageing phenotypes. Surprisingly, only ATM (Ataxia Telangiectasia Mutated) protein deficiency caused early leaf ageing, resulting from an over-accumulation of double-strand breaks [124]. This raises the question: does DNA repair-redundancy in plants mitigate the effect of single gene knock-outs, or does the ATM mutant affect ageing as a secondary result of gross genome instability? These studies indicate that DNA repair is not a major player in *Arabidopsis* ageing regulation. Nevertheless, the relationship between DNA repair and plant ageing may be complex and could differ between plant species; thus, further study into this area is warranted.

### Hormones are major plant growth regulators and govern ageing

Plant hormones are one of the most important internal factors that regulate plant development and influence ageing. Over the past few decades, hormones have been studied extensively, and excellent reviews have been published on the role of hormones that positively or negatively regulate plant ageing [17, 125–129]. There is ample genetic evidence confirming the role of ethylene, JA, SA and ABA as positive and GAs as negative regulators of ageing [17, 130–132].

However, the role of auxin in plant ageing is less understood and contradictory. Some reports describe auxin as a promoter of ageing, whereas others consider it to delay ageing. For instance, abundance of the biologically active auxin hormone indoleacetic acid (IAA) was found to increase during age-dependent leaf senescence [133], suggestive of a role in senescence induction. Auxin can also induce the biosynthesis of ethylene, which is a strong senescence-inducing hormone [134]. Furthermore, a mutation in the *SAUR36* gene, which mediates auxin-induced leaf senescence, showed delayed plant ageing [135]. Likewise, mutations in *Arabidopsis* *ARF1* and *ARF2* (*Auxin response factor*) delayed leaf senescence progression in the mutant plants [136, 137]. However, treatment of *Arabidopsis* leaves with auxin decreased expression of senescence marker gene *SAG12* [138], and *35S*:*YUC6* transgenic plants, which exhibit increased IAA abundance, showed extended leaf longevity [139]. Thus, a dual role of auxin has been observed in plants’ ageing, which does not contradict the transcriptomic studies.

Transcriptomic studies do not provide clear hypotheses as to whether cytokinins are positive or negative regulators of ageing. However, molecular genetic analyses clearly demonstrate that the cytokinin hormone delays ageing in plants. For example, various transgenic plants introduced with the *IPT* gene (*Isopentyl transferase*, a gene involved in cytokinin biosynthesis) under various promoters showed delayed senescence [140–143]. Also, the cytokinin level in old tobacco leaves was lower than that in young leaves [144]. Furthermore, the exogenous application of cytokinin on numerous monocot and dicots plants is reported to delay senescence by preventing chlorophyll breakdown and inhibiting protein degradation [127, 144–146].

Thus, functions of major plant hormones in regulating plant ageing as predicted by transcriptomics studies, can be largely confirmed by molecular genetic experiments, although exceptions, notably the roles of auxins and cytokinin, exist.

## Primary metabolism as drivers of ageing

Understanding ageing—and ultimately being able to control it—is one of the holy grails of biological sciences. Nevertheless, progress in this area is painfully slow, and past molecular genetic analyses have provided important, but sometimes confusing results. Recent advances in systems biology have allowed us to develop new, testable hypotheses and, crucially, many of these have already been tested. A picture is emerging where basic biological processes are master controllers of ageing (Fig. 3). In plants, hormones have long been demonstrated to play important roles in ageing and this view has not changed, with ethylene, ABA, JA, SA as ageing-inducing hormones and GA and cytokinin as ageing repressors. Nevertheless, all plant hormones fulfil multiple roles throughout plant development, which may explain discrepancies between predictions based on transcriptomics and molecular genetic analyses. The systems biology approaches furthermore strongly support a role for oxidative stress in ageing. In addition, core cellular processes appear to be fundamental to ageing, possibly feeding into oxidative stress metabolism (Fig. 3).

**Fig. 4:**
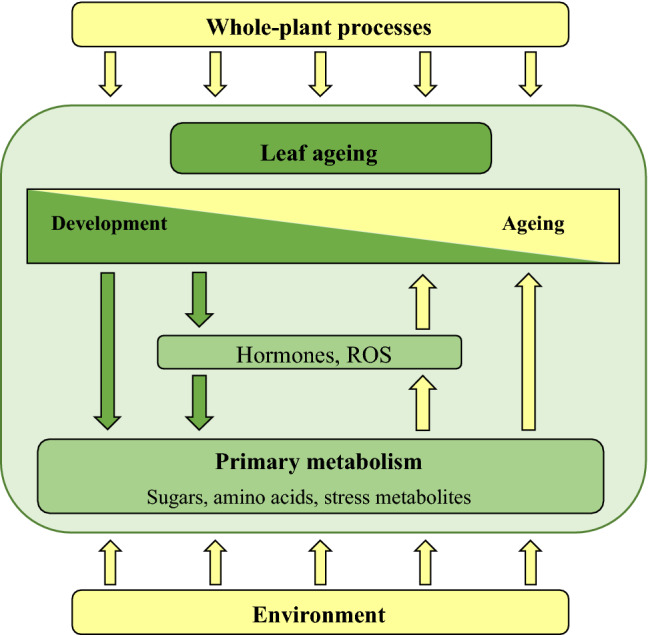
Speculative model proposing that leaf development affects primary metabolism and that this inevitably leads to leaf ageing. Plant hormones and reactive oxygen species (ROS) play a central role in this process as they are affected by development and primary metabolites. The speed of ageing, but not leaf ageing itself, can be modulated by environmental stress and whole-plant processes, such as reproduction in monocarpic and season in deciduous plants

Expanding on this idea, we propose that changes in primary metabolite abundances, caused by leaf developmental processes, are driving leaf ageing. The concept that leaf development is intimately linked with leaf ageing is not new, but here we posit the idea that primary metabolism constitutes this link (Fig. 4). The figure highlights that leaf development inherently affects primary metabolism, directly and through the action of hormones and ROS. For example, the end of cell division and expansion, changing source-sink partitioning and a decrease in photosynthetic efficiency changes the abundances of sugars and ROS; decreased metabolites contributing to antioxidant activity further increases ROS abundance. These changes in primary metabolites then drive ageing, likely at least partly through modulation of ROS abundance. The figure also shows that leaf ageing is not a rigid process: environmental factors like stress and whole-plant processes such as monocarpic senescence can modulate ageing of individual leaves. The model thus hypothesises how leaf ageing is hard-wired to developmental processes and is consistent with decades-long research, where no mutant has been found that does not age, and many ageing mutants show alterations in development.

Plant ageing is still far from being understood. What can be done to move this field forwards? Since ageing is so closely integrated into development—or perhaps they can even be considered one and the same—it will be difficult to separate developmental from ageing processes. Nevertheless, the model presented in Fig. 4 makes two predictions that may help design experiments that can test the model: (1) ageing is a continuous process and if early ageing is affected, then the timing of the final leaf ageing stage, leaf senescence, should also be affected; (2) primary metabolism affects ageing throughout leaf development. Most research in plant ageing has been done on senescence, which is the final stage of ageing. Therefore, increased emphasis should be placed on the early stages of ageing, while the onset of leaf senescence could still be used to estimate total leaf longevity. Furthermore, transient disruptions in primary metabolism may not affect whole plant development, while the model predicts that this would affect leaf ageing and lifespan. Here, multi-omics approaches, including metabolomics, can detect patterns that may not be detected using targeted analyses. It may be more challenging to study the early developmental stages because they are less defined, and many growth processes take place at the same time. However, systems biology analyses can tease out individual processes and molecular genetic approaches can robustly test hypotheses. We also would like to encourage researchers to test the model in diverse plant species, including perennials, to determine how distinct plant species modulate general ageing processes to benefit survival. We are looking forward to further progress in this age-old scientific field.

## Data Availability

All data generated and analysed during this study are included in this manuscript.
